# Transcriptomic Evidence Reveals the Molecular Basis for Functional Differentiation of Hemocytes in a Marine Invertebrate, *Crassostrea gigas*

**DOI:** 10.3389/fimmu.2020.00911

**Published:** 2020-05-27

**Authors:** Fan Mao, Nai-Kei Wong, Yue Lin, Xiangyu Zhang, Kunna Liu, Minwei Huang, Duo Xu, Zhiming Xiang, Jun Li, Yang Zhang, Ziniu Yu

**Affiliations:** ^1^CAS Key Laboratory of Tropical Marine Bio-resources and Ecology and Guangdong Provincial Key Laboratory of Applied Marine Biology, South China Sea Institute of Oceanology, Chinese Academy of Science, Guangzhou, China; ^2^Southern Marine Science and Engineering Guangdong Laboratory (Guangzhou), Guangzhou, China; ^3^Innovation Academy of South China Sea Ecology and Environmental Engineering, Chinese Academy of Sciences, Guangzhou, China; ^4^Department of Infectious Diseases, Shenzhen Third People's Hospital, The Second Hospital Affiliated to Southern University of Science and Technology, Shenzhen, China

**Keywords:** oyster, functional differentiation, granulocytes, Cdc42, Fos

## Abstract

Hemocytes play unequivocally central roles in host immune defense of bivalve mollusks, though the exact mechanisms underlying their functional differentiation are only partially understood. To this end, granulocytes and hyalinocytes were sorted via flow cytometry from hemocytes of the Pacific oyster *Crassostrea gigas*, and consequently quantitative transcriptomic analysis revealed a striking array of differentially expressed genes (DEGs), which were globally upregulated in granulocytes, dedicating to functional differentiation among oyster hemocytes. Our network of DEGs illustrated actively engaged signaling pathways, with Cdc42/Cdc42l being a core regulator of pathway network, which was validated by a dramatically reduced capacity for hemocyte phagocytosis in the presence of Cdc42 inhibitors. Additionally, a number of transcription factors were identified among DEGs, including ELK, HELT, and Fos, which were predominantly expressed in granulocytes. The AP-1 transcription factor Fos was confirmed to facilitate functional differentiation of hemocytes in an assay on binding to target genes by the AP-1 binding site, consistent with downstream phagocytosis and ROS production. Importantly, Cdc42/Cdc42l were also regulated by the expression of Fos, providing a possible regulatory mechanism-guided hemocyte functional differentiation. Findings in this study have bridged a knowledge gap on the mechanistic underpinnings of functional differentiation of hemocytes in a marine invertebrate *C. gigas*, which promise to facilitate research on the evolution of immune defense and functional differentiation of phagocyte in higher-order and more recent phyla.

## Introduction

Marine invertebrates are intrinsically useful reductionist models for investigating host defense primarily based on innate immunity. In bivalve mollusks, an open circulatory system is populated by hemocytes, which patrols between hemal sinus and soft tissues. These immunologically plastic cells excel at performing a diverse range of cellular functions including phagocytosis of invading pathogens, encapsulation of bulky invaders, enzymatic digestion and transport of nutrients, and biosynthesis and secretion of humoral factors (1–3). Hemocytes have been variously classified in terms of their morphology, cytochemistry, and function. Other methods such as flow cytometry (4, 5), density-gradient centrifugation (6), and immunostaining of cell surface proteins (7, 8) have also been proposed to characterize hemocyte subtypes. Despite some controversies, it is generally agreed that hemocyte subtypes in mollusks consist of two principal cell types in the hemolymph: granulocytes and hyalinocytes (also known as agranulocytes) (9, 10). However, hemocyte subtypes have been further divided into three, four, and even more different populations based on different parameters applied by researchers for various bivalve species (5, 11–13). Consequently, it is often difficult to compare or generalize findings between studies. In mollusks, as is true in many other invertebrates, the presence of a hemopoietic organ is not the norm; hemocytes may instead be formed in various ways. For example, spontaneous mitosis of hemocytes increases during circulation in hemolymph vessels, sinuses, and soft tissues (14–16). This raises the possibility of observing plasticity during various stages of hemocyte maturation, rather than simply categorizing cells into distinct subtypes (17–19).

Conventionally, the presence or absence of cytoplasmic granules as an intuitive criterion has inspired the classification of hemocytes into granulocytes and hyalinocytes, as mentioned above. These two cell types have been reported in many species, including *Mytilus edulis* (20), *Tapes philippinarum* (21), *Biomphalaria glabrata* (22), *Ruditapes decussatus* (23), and *Crassostrea gigas* (24). Of the two, hyalinocytes are cells that are smaller and harbor few or no cytoplasmic granules. They can be morphologically further divided into two subclasses: small hyalinocytes with large nuclei and large hyalinocytes with small nuclei and large cytoplasm (14). Granulocytes are characterized by their ability to efficiently phagocytize microorganisms, generate reactive oxygen species (ROS) and express hydrolytic enzymes that contribute to intracellular killing (25–28). In general, granulocytes have a greater phagocytic capacity than hyalinocytes. To this date, however, the molecular mechanisms underlying the functional differentiation of hemocytes remain largely enigmatic.

The granulocytes are evolutionary analogous of macrophage and neutrophil in mammals, which could be functionally differentiated from circulating monocytes in the bloodstream after infection or vaccination circulate (29). Combined action of critical transcription factors can determine the expression of myeloid-specific genes and the generation of macrophages (30). Moreover, transcription factors are anticipated to play pivotal roles in marshalling proliferative and differentiated signals into genetic programs, determining the cell fate, growth stimulation, functional activation, and lineage-specific evolution (31–33). It has been proposed that specific transcription factor activity is mandatory for multiple lymphoid lineages, such as *Nfil3* and *Tcf1* for innate lymphoid cell (ILC) development (34) and *Ets* family transcription factors for NK cell development (35, 36). It is also known that PI3K/AKT signaling cascade plays a vital role in the synthesis of granules during stressful stimulation (37).

Previous studies in oyster have shown that granules in granulocytes react for acid phosphatase, which is a typical characteristic of lysosomes and participates in intracellular digestion of particles, widely accepted as markers of functional differentiation of hemocytes (38). However, how granules and proteolytic enzymes arise to generate functional hemocytes is at best incompletely understood in oyster. The Pacific oyster, *C. gigas*, one of the most prominent aquacultural mullosk species with global distribution, depends on innate immunity for anti-infective defense. A wide range of microorganisms can be phagocytized and cleared by *C. gigas* hemocytes. With the advent of technological improvements, flow cytometry (FACS) has been applied to analyze cellular properties in hemocytes including cell types and their frequency (4, 5, 39). In this study, we attempted to investigate the potential determinants of plasticity leading to the functional differentiation between hyalinocytes and granulocytes. *C. gigas* hemocytes were isolated and analyzed by FACS coupled to quantitative transcriptomics analysis, which provided a new modality for comparing differential genes in the two hemocytes subtypes. A panel of differentially expressed genes (DEGs) of high interest including key transcriptional factors was identified in this study. A network on the basis of DEGs was constructed to illustrate the relationship between actively engaged signaling pathways and core components implicated in functional differentiation of hemocytes. Additionally, the potential significance of transcriptional factors regulating functional activity of hemocytes was further scrutinized via knocking down expression of the specific genes *in vivo*.

## Materials and Methods

### Animal Collection and Maintenance

The Pacific oysters, *C. gigas* (2 years old with an average shell length of 100 mm), were obtained from Qingdao, Shandong Province, China, and maintained at 22–25°C in tanks with re-circulating seawater before experiments. Treatment-naïve and pathogen-free oysters were chosen for experiments, independently of their genetic background. Oysters were fed twice daily on *Tetraselmis suecica* and *Isochrysis galbana*. They were held for 2 weeks prior to experimentation.

### Hemocyte Preparation

To collect hemocytes, the oyster shell was carefully opened and all mantle fluid was drained. Approximately 1 ml of hemolymph per oyster was sampled from adult *C. gigas* individuals by using a 1-ml syringe with a 25-mm needle inserted into the pericardial cavity. Immediately, hemolymph was placed on ice to prevent hemocyte aggregation, followed by centrifugation at 1,500 × *g* at 10°C for 10 min. Cell pellets containing hemocytes were removed and suspended in 1 ml of cell protection medium, as previously reported (32). Samples were kept on ice until used for experiments.

### Sorting of Granulocytes and Hyalinocytes

Hemolymph samples were analyzed and sorted by using a BD Biosciences FACSCanto II flow cytometer (Becton Dickinson, USA). For each group, 10 oysters were randomly grouped for hemocyte preparation and cell sorting. A total of four groups of hemocytes (R1, R2, R3, and R4) were used in sorting granulocytes and hyalinocytes. After preparation as mentioned above, hemocytes were sorted on the basis of their cellular granularity and cell sizes in flow cytometry by using CellQuest program. For each sample, 20,000 cells were sorted.

### Imaging of Granulocytes and Hyalinocytes

Sorted cell subpopulations were imaged by light microscopy and transmission electron microscopy (TEM). Briefly, granulocytes and hyalinocytes were placed onto glass slides, and their observations were carried out under a light microscope (Nikon E100). Additionally, hemocytes were also prepared for examination under a transmission electron microscope. Briefly, sorted granulocytes and hyalinocytes were mixed with 5% glutaraldehyde fixative solution, followed by centrifugation to remove supernatant. Then, cells were prefixed again with 2.5% glutaraldehyde fixative solution at 4°C, followed by mixing in 1% osmium tetroxide and dehydration in ethanol. Subsequently, cells were embedded in Epon epoxy resin, and left to harden at 60°C. Ultrathin sections were prepared on a Leica EMuC7 and post-stained with 0.5% aqueous uranyl acetate, and then exposed to lead citrate. Finally, electron micrographs of the sections were acquired under a Hitachi HT7700 transmission electron microscope.

### Flow Cytometry Analysis

To compare phagocytic abilities of granulocytes and hyalinocytes, FITC-labeled bacteria (*Vibrio parahaemolyticus* E151) were added to hemocytes (2.5 × 10^5^ cells) cultured in a 24-well plate for 15 min, in a 50:1 ratio (hemocytes/bacteria). Trypan blue (1.2 mg/ml) was used to quench surface-bound FITC-labeled bacteria. Then, hemocytes were washed three times with Tris buffer (50 mM at pH 8.0) and re-suspended in PBS supplemented with 15% EDTA. Subsequently, flow cytometry analysis was performed to quantify hemocyte subpopulations. Granulocytes and hyalinocytes were gated by using at least 10,000 events per sample based on cellular granularity and cell sizes. To compare capacities for ROS generation in granulocytes and hyalinocytes, hemocytes were collected and stained with 5 μM dichlorodihydrofluorescein diacetate (DCFH, prepared with PBS buffer) in plasma at room temperature for 60 min. Then, hemocytes were washed with pre-warmed PBS to remove excess probe, followed by data acquisition by flow cytometry. At least 10,000 events per sample were collected for comparison on ROS production between granulocytes and hyalinocytes. Flow cytometry data were analyzed using FlowJo software, and statistical difference was calculated by Student's *t* test for triplicated data of granulocytes and hyalinocytes.

### Library Preparation and RNA Sequencing

RNA library was prepared using the REPLI-g WTA single cell kit (150063, Qiagen, German). Briefly, a single cell sample (containing 1,000 cells) is lysed efficiently within 5 min. Following cell lysis, gDNA was removed prior to WTA process. Poly-adenylated transcripts were amplified by using oligo dT primers. Synthesized cDNA was ligated using a high-efficiency ligation mix. Ligated cDNA was amplified utilizing MDA technology, with novel REPLI-g SensiPhi DNA polymerase, in an isothermal reaction lasting 2 h. Consequently, amplified cDNA was examined for its suitability for RNA sequencing. After construction of a cDNA library, Qubit 2.0 and Agilent 2100 were used to detect concentrations of the library. Q-PCR method was used to accurately quantify effective concentrations of the library to ensure quality of the library. Subsequently, high-throughput sequencing was performed with HiSeq2500, and sequencing reading length was SE50. Original image data files obtained from Illumina HiSeq2500 high-throughput sequencing platforms were transformed into raw data or raw reads by the base calling. Results were stored in FASTQ file format, which contained information of sequenced transcripts and their corresponding sequencing quality information. Four biological replicates of transcriptomic sequencing were obtained in each case, and all raw data were deposited in the NCBI Sequence Read Archive database under the accession number PRJNA591303. Information on sequencing data is as presented in Table S1.

### Bioinformatics in Transcriptomic Analysis

Sequence alignment and subsequent analysis were performed using the designated *C. gigas* genome as a reference genome using TopHat2 software (40). Information of alignment efficiency statistics is as presented in Table S2. Bowtie (41) was used for comparison and transcript expression levels were estimated according to comparison results in conjunction with information from Cufflinks/RSEM (42). Finally, RPKM (41) values were used to gauge the expression abundance of corresponding Unigenes, for calculating and comparing gene expression differences between individual samples. Absolute values of log_2_ (fold change) > 1 and FDR (false discovery rate) value < 0.01 were set as threshold parameters to determine DEGs in granulocytes and hyalinocytes in each group (Table S3). Then, differential combinatorial analysis on DEGs in each group was performed to determine common DEGs. BLAST software was used to sequence the DEGs with NR, Swiss-Prot, GO, COG, and KEGG to obtain annotation information on DEGs as shown in Table S4. Results from KEGG analysis are as presented in Figure S1. Factoextra R Package was applied in principal component analysis (PCA). RNA expression levels of DEGs in different *C. gigas* tissues were analyzed based on *C. gigas* transcriptomic data (43).

### Protein Network Mapping

STRING server (http://string.embl.de) was used to predict interacting partners in protein—protein interactions. We compared protein—protein interactions using the protein databases of *Danio rerio* and *Drosophila melanogaster*, which are species considered evolutionarily relevant to oysters. Further, the *D. rerio* protein database was found to be more suitable for constructing protein interactomes of oyster hemocytes DEGs, based on the number and similarity of aligned proteins. Networks were constructed based on proteins predicted from DEGs (*p* <0.05) between granulocytes and hyalinocytes. Disconnected nodes were hidden. The entire networks are available for interactive visualization of protein interactions in Cytoscape session file.

### Inhibition Assay on Cdc42 Protein

In addition, flow cytometry was also conducted to explore the functional roles of Cdc42 protein in granulocytes and hyalinocytes using inhibitors of Cdc42 (MLS-573151: Cat. no. C4738; Casin, Cat. no. B6103. APExBIO, USA). The dosage of the inhibitors were determined on the basis of published literatures (44–46). First, oyster hemocytes were harvested from the pericardial cavity and seeded into a 24-well plate for 15 min. This was followed by addition of inhibitors at appropriate concentrations (Casin at 10 μM and MLS-573151 at 50 μM) for 15 min. Ten microliters of FITC-labeled beads (Sigma, USA, 90305) and FITC-labeled bacteria (*V. parahaemolyticus* E151) were added to hemocytes (2.5 × 10^5^ cells) in a 50:1 ratio (hemocytes/bacteria), which were incubated at room temperature for 15 min. Upon establishment of phagocytosis (15 min), all samples were incubated with Trypan blue (1.2 mg/ml) to quench surface-bound FITC-labeled beads or bacteria, and further washed twice with PBS buffer supplemented with 15% EDTA to remove non-phagocytic beads or bacteria. Finally, flow cytometry analysis was performed to quantify phagocytosis-related fluorescence in oyster hemocytes. Gates were applied to define granulocytes and hyalinocytes. Cell phagocytosis was monitored using at least 10,000 event per sample. Data was analyzed with FlowJo software, and statistical difference was determined by one-way ANOVA for triplicated data of granulocytes and hyalinocytes. Moreover, the suppressive effect of the Cdc42 inhibitors in oyster was validated by test the expression level of Wiskott–Aldrich Syndrome Protein (WASP), which is considered as the core effector of Cdc42.

### *In vivo* RNAi Assay

To clarify the roles of ELK, HELT, and Fos in generation of functional granulocytes, the genes were knocked down *in vivo via* dsRNA-mediated RNA interference. The primers used to synthesize dsRNA are as shown in Table 1. ELK, HELT, Fos, and a GFP cDNA fragment (negative control) were amplified with primer pairs of T7 promoter overhangs in the Promega RiboMAX™ Express RNAi System. PCR products were used as templates to synthesize dsRNA according to the manufacturer's instructions. For this experiment, oysters were randomly divided into four groups: ELK-interference group (iELK), HELT-interference group (iHELT), Fos-interference group (iFos), and control group (iGFP). Each oyster was injected with 50 μg dsRNA and individuals from each group were randomly selected for collection of hemocytes. iGFP indicated the control group that was injected with equal amount of GFP dsRNA. All the experimental groups were compared with the iGFP group to calculate the transcriptional effects of these transcription factors on target genes. RNAi efficiency was ascertained by two independent quantitative real-time PCR. In each independent experiment, three samples were applied to detect the gene mRNA expression profile. Genes related to phagocytosis and ROS production were determined by qRT-PCR (quantitative real-time PCR) after knockdown of ELK, HELT, and Fos.

**Table 1:** Primers used in this study.

**Name of Primers**	**Sequence (5^**′**^-3^**′**^)**
dsHELT-F	GGATCCTAATACGACTCACTATAGGTAGTGAATGGTTTTC TTCGGAC
dsHELT-R	GGATCCTAATACGACTCACTATAGGAGAATAAGCCGGT GAAAGGA
dsTranF-F	GGATCCTAATACGACTCACTATAGGCAAACCGCCTTATTA TTGAAAC
dsTranF-R	GGATCCTAATACGACTCACTATAGGAAGAGGAGGGTCCG TTAGTG
dsElk3-F	GGATCCTAATACGACTCACTATAGGGGACACCAATGTGA CGCTAT
dsElk3-R	GGATCCTAATACGACTCACTATAGGAGGGCAGATGCTT CAGTTTT
β-actin F	AAGATATTGCAGCTTTAGTCGT
β-actin R	TTCTGTCCCATACCAACCAT
qHELT-F	GAGCAGACTTCACACAAGATCA
qHELT-R	CTATTTTCAAAGCGTAAACCAA
qElk3-F	AATGCCATCGCCTCCTCTTC
qElk3-R	ATGGAGACTTTCGGACTTGGA
qTranF-F	ACCCGCACCACCATCCTC
qTranF-R	AGATTTGTGGGCACCGACTG
qRab11-F	GGAAACAGGCAAAGGCAAGA
qRab11-R	AATGACGAGTCCAGCAAGGG
qCD63-F	TTAGAAATCTCGGCGGGAATA
qCD63-R	GACACTCCACAACATTTGAACTCTT
qRho1-F	GGAACCAGTCAAATCGCAAGA
Rho1-R	GAGCTGCCCTAGTGGCTGTT
qLAMP-F	TCTAGAACTGATATTATGTA
qLAMP-R	AATGTTTTCATTGTGCATTA
qSOD-F	TACGAAAACTCCATGCATT
qSOD-R	TTCATTATGGTACAGTT
qFerritin1-F	AACGGGAACATGCTGAA
qFerritin1-R	TTGCTCCTCCAAGTAGT
qFerritin2-F	AGTTGATGAAATACCAGA
qFerritin2-R	TCAGAAATCTCCTTGAT
qNd5-F	CAATAAAGACGTAACTTTT
qNd5-R	TGGATGGGATAGTTACAAAT
qCdc42-F	GACACGGCTGGAAAGGAGG
qCdc42-R	TAAATGGCGTGTTTGGCACA
qCdc42l-F	GTGGAGAAGTTCTGGGTCAATGA
qCdc42l-R	CTATCCCGTCGCCCGTTTT
qCYTB-F	AGGGCTTAGCCTAGCTCACGT
qCYTB-R	CGCCAACAGCAATGCAAAC

### Expression Profile Analysis by qRT-PCR

qRT-PCR was conducted using a LightCycler 480 (Roche) with a reaction volume of 10 μl containing 1 μl of template cDNA, 5 μl of 2 × SYBR Green Mix, 0.5 μl of each primer (10 pmol/μl), and 3 μl of PCR-grade water. The qRT-PCR cycle program consisted of one cycle of 95°C for 1 min, followed by 40 cycles of amplification at 95°C for 15 s, 55°C for 15 s, 72°C for 20 s, and 85°C for 20 s. The relative expression of the genes was calculated using the 2^−ΔΔCT^ method. All experiments were performed in triplicate using β-actin mRNA as an internal control. All data are represented in terms of relative mRNA levels.

### Prediction of Transcription Factor Binding Sites

PROMO prediction software (http://alggen.lsi.upc.es/) was used to identify the transcription factor binding sites of some transcription factors (ELK, HELT, and Fos), phagocytosis-related genes (LAMP, Rab11, CD63, Cdc42, Cdc42l, and Rho), and ROS production-related genes (SOD, ferritin1, ferritin2, Nd5, and CytB1).

### Dual-Luciferase Reporter Assay

Subsequently, promoter sequence of Cdc42 [−485, −1] bp and Cdc42l [−349, −1] bp were cloned into pGL3 basic plasmid for dual luciferase reporter assay. For DNA transfection, cells were seeded and allowed to grow to about 70% confluence, followed by plasmid transfection with Viafect reagent (Promega, USA) according to the manufacturer's recommendations. For dual-luciferase reporter assays, HEK293T cells were transiently co-transfected with pRL-TK vector (20 ng/μl), luciferase reporter vectors (AP-1 reporter Luc, Cdc42-promoter Luc, and Cdc42l-promoter Luc, 300 ng/well), and recombinant plasmid pcDNA (0, 150, and 300 ng/well). Cdc42-promoter and Cdc42l-promoter reporter vectors were constructed by cloning the promoter sequence of Cdc42 and Cdc42l to pGL3-basic plasmid. AP-1 reporter vectors used in this study were previously constructed in our laboratory. A pRL-TK vector (Promega, USA) was used as an internal control. Cells were transfected in serum-free culture medium for 4–6 h, followed by replacement of culture medium with fresh complete MEM.

At 48 h post-transfection, HEK293T cells in 48-well plates were washed with PBS twice and lysed. Firefly and Renilla luciferase activity was measured in a luciferase reporter assay system (Promega, USA) according to the manufacturer's instructions. Relative luciferase activity was calculated by normalization to Renilla luciferase values. Experimental results are expressed as fold changes relative to the empty vector control. All results are represented as the mean ± SEM. Statistical significance was analyzed with GraphPad software.

### Electrophoretic Mobility Shift Assay (EMSA)

A biotin-labeled AP-1 probe was designed (GeneWiz, USA) and used to perform EMSA. Detection of biotin-labeled DNA by chemiluminescence was performed based on an established protocol (Thermo Fisher, USA, 20148). Briefly, biotin-labeled double-stranded DNA was incubated with hemocyte extract proteins, and then non-denatured gel electrophoresis was performed. The DNA was then transferred rapidly (30 min) to a positively charged nylon membrane for purple diplomatic conjugation, which then proceeded directly to detection.

## Results

### Cell Typing and Morphological and Functional Characterization of Hemocyte Subtypes

**Figure 1:**
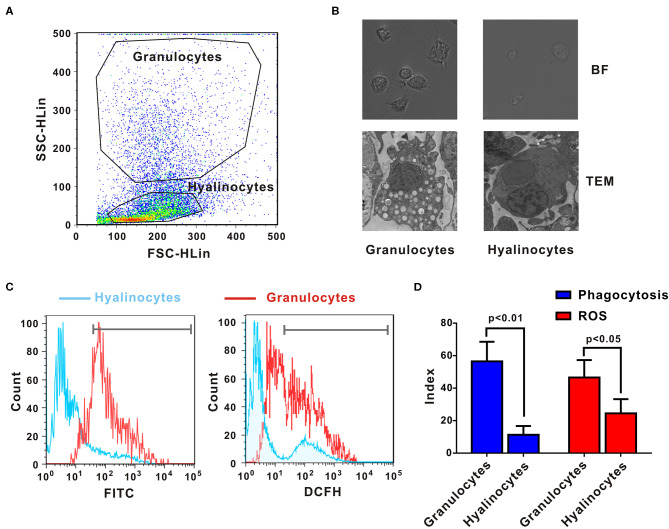
Morphological and functional characterization of *C. gigas* granulocytes and hyalinocytes. **(A)** Flow cytometry analysis was conducted to define hemocyte subpopulations. Granulocytes and hyalinocytes were gated, respectively. **(B)** Granulocytes and hyalinocytes were imaged by light microscopy and TEM. **(C)** Flow cytometry analysis on the differences between granulocytes and hyalinocytes in terms of phagocytosis (*left panel*) and ROS production (*right panel*). The blue line here represents the functional parameters of hyalinocytes, and the red line represents those of granulocytes. **(D)** Data analysis was performed by using GraphPad 5 software and vertical bars represent mean ± SEM (*n* = 3). Phagocytosis index was represented in the left panel and ROS production was represented in the right panel.

*Crassostrea gigas* hemocytes are professional immune effector cells adept at phagocytosis and killing of bacteria. For cell sorting, hemolymph from 10 oysters was analyzed by flow cytometry, in which two cell populations with distinguishable complexity (slide scatter, SSC) and continuous size (forward scatter, FSC) were found (Figure 1A). A representative section with low SSC constituted an agranular population, corresponding to hyalinocytes. Another representative section with high SSC was a high-granularity population, corresponding to granulocytes. Then, these two subpopulations of oyster hemocytes were sorted for RNA extraction and transcriptomic analysis, which were also confirmed based on morphological features under a light microscopy (Figure 1B). Ultrastructural analysis by TEM revealed detailed morphological traits (as shown in Figure 1B). Evidently, *C. gigas* hyalinocytes were characterized by few or no granules in the cytoplasm, which were packed with vacuoles. In contrast, *C. gigas* granulocytes were characterized by an eccentric nucleus and numerous cytoplasmic granules.

Phagocytic activities of different hemocyte subpopulations were examined by flow cytometry analysis, as shown in Figure 1C (*left panel*). After incubation with FITC-labeled bacteria, phagocytic indices of granulocytes and hyalinocytes were quantified. Higher fluorescence intensities were detected in granulocytes, which could ingest more bacteria to emit stronger fluorescence. The hyalinocyte population reported weaker fluorescence intensities and low phagocytosis rates. Furthermore, the phagocytic index was ~58 ± 8.6% in granulocytes and 12 ± 2.4% in hyalinocytes. Phagocytic capacity of granulocytes was significantly higher than that of hyalinocytes (*p* < 0.01) as shown in Figure 1D (*blue panel*). Spontaneous production of ROS during oxidative bursts was also examined with the probe DCFH in oyster hemocytes by flow cytometry, as shown in Figure 1C (*right panel*). For oysters under resting condition, basal levels of ROS production of granulocytes were significantly higher than those in hyalinocytes (*p* < 0.05), as shown in Figure 1D (*red panel*).

### Transcriptomic Analysis on DEGs in Granulocytes and Hyalinocytes

**Figure 2:**
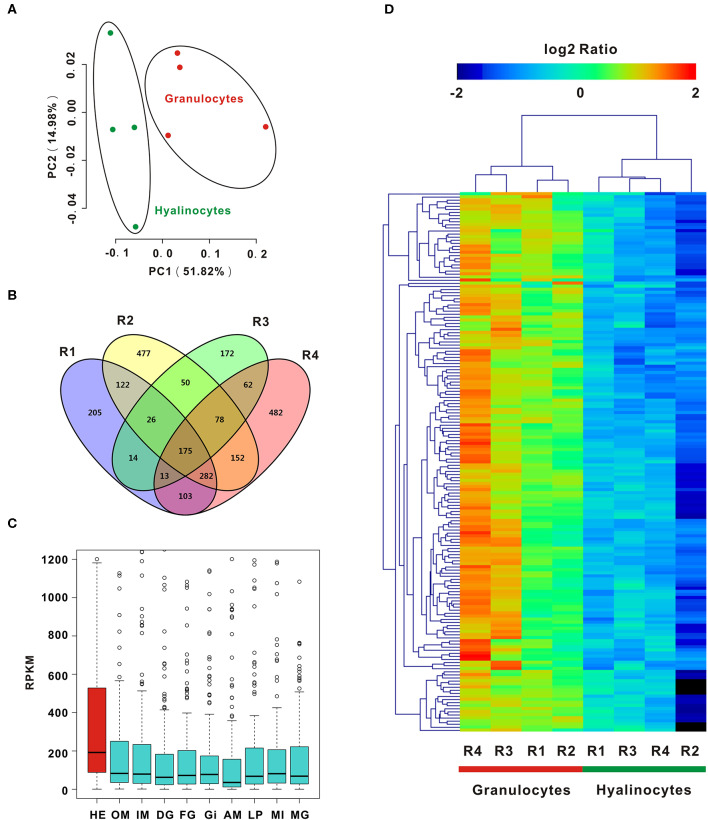
PCA and DEGs analysis of *C. gigas* granulocytes and hyalinocytes. **(A)** Scores plot of principal components analysis on *C. gigas* hemocyte subpopulations (granulocytes and hyalinocytes). PC1 and PC2: principal component 1 and principal component 2. Each point represents a metabolite profile of a biological replicate. **(B)** DEGs were first explored in each group for comparison between granulocytes and hyalinocytes. Venn diagram was constructed to determine common DEGs in the four groups (R1, R2, R3, and R4). **(C)** Tissue distribution of DEGs in *C. gigas*. Red box represents the high expression of DEGs in hemocytes. HE, hemocytes; OM, outer mantle; IM, inner mantle; DG, digestive grand; FG, female gonad; GI, gill; AM, adductor muscle; LP, labial palps; MI, mixture of adult tissues; MG, male gonad. **(D)** Data for relative expression levels of genes were obtained by DEGs data. Colors from red to blue indicate range of log_2_ ratio values (in descending order); red color indicates high expression level and blue color indicates low expression level.

To enable analysis on the molecular determinants of functional differentiation, cell subpopulations were first sorted based on cell size and granularity, given a lack of granulocyte-specific or hyalinocyte-specific antibodies. A wide range of granulocyte and hyalinocyte transcriptome libraries were constructed from four groups mentioned above and sequenced by using a high-throughput RNA-seq platform. Next, PCA was conducted for whole datasets. PCA results for all surveyed genes in granulocytes and hyalinocytes suggested that genes in datasets obtained from the same cell subpopulations clustered tightly; different cell subpopulations were discriminated at different groups, indicating that there is a high degree of homogeneity in gene expression pattern in each hemocyte type (Figure 2A). The profiles revealed disparity in gene expression between oyster granulocytes and hyalinocytes.

In order to further clarify the differences between granulocytes and hyalinocytes at the transcriptomic level, analysis was performed to screen for DEGs under different biological conditions. Biological replicates (R1, R2, R3, and R4) were established as described in Materials and Methods. We first screened for DEGs of granulocytes and hyalinocytes in the biological replicates. In between-group comparisons, genes having a FDR value < 0.01 and fold change (FC) ≥ 2 found by DESeq were assigned as DEGs. Then, we screened for common DEGs among R1, R2, R3, and R4, after determining that 175 genes were expressed at significantly different levels (Figure 2B). We also validated the expression level of key DEGs via QPCR, and the result was displayed in Figure S1. In addition, we analyzed the mRNA expression level of DEGs in different tissues including hemocytes, outer mantle, inner mantle, digestive grand, female gonad, gill, adductor muscle, labial palps, mixture of adult tissues, and male gonad (Figure 2C), based on the published *C. gigas* transcriptomic data (43). Predominant transcript expression patterns in hemocytes were established. Subsequently, a heatmap of DEG expression levels in granulocytes and hyalinocytes was constructed (Figure 2D). The correlation distance of DEGs were analyzed and presented in Figure S3, in which stronger heterogeneity was observed in granulocytes rather than hyalinocytes. Surprisingly, all common DEGs in the biological replicates were upregulated in granulocytes, suggesting more complex regulation network in the granulocytes than hyalinocytes.

### Cdc42 Regulatory Network Revealed by Analysis on DEG Pathway Networks

**Figure 3:**
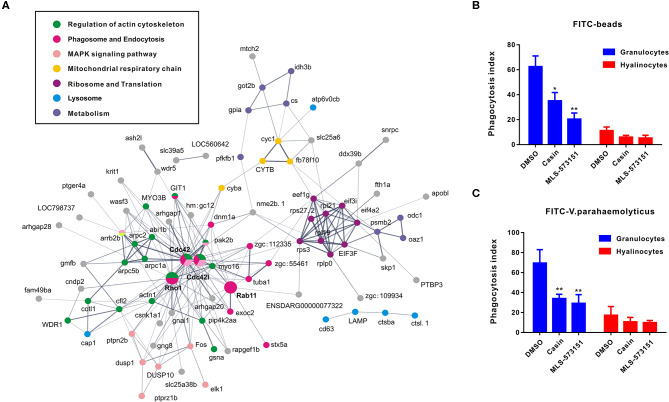
Networks based on DEGs implicating central roles of Cdc42 in granulocyte phagocytosis. **(A)** Networks are represented here schematically. Nodes represent proteins. Edges represent interactions between proteins. Different colors represent different pathways and proteins. **(B,C)** Phagocytosis index of FITC beads **(B)** or FITC-labeled bacteria (*V. pa*) **(C)** by *C. gigas* granulocytes and hyalinocytes in the presence of Cdc42 inhibitors (casin and MLS-573151). Blue panel represents granulocytes and red panel represents hyalinocytes. *p < 0.05; **p < 0.01.

As an attempt to shed light on the possible functions of significantly expressed genes identified in transcriptomic analysis, KEGG pathway analysis was performed to pinpoint pathways that contributed importantly to functional differentiation of hemocytes (Figure S2). In this aspect, our results implicated 10 overrepresented pathways (*Q* value < 0.05), among which “regulation of actin cytoskeleton,” “Fc gamma R-mediated phagocytosis,” “leukocyte transendothelial migration,” and “pathogenic *Escherichia coli* infection” are generally related to granulocytes formation. Furthermore, to delineate the dynamic details of oyster hemocytes during functional differentiation, we mapped out a DEG pathway network based on predicted proteins corresponding to all 175 DEGs in the transcriptomic analysis. Essentially, these genes could primarily be organized into several categories, namely, “regulation of actin cytoskeleton,” “phagosome and endocytosis,” “MAPK signaling pathway,” “mitochondrial respiratory chainm” “ribosome and translation,” “lysosome,” and “metabolism,” Meanwhile, these genes formed a regulatory network resolving around Cdc42 (cell division control protein 42) as a core member (Figure 3A). Cdc42 is a central protein implicated in the regulation of cell cycle and is known to critically impact a variety of signaling events and cellular processes in many organisms (47).

In order to verify what extent Cdc42 was involved in regulating hemocyte function, two Cdc42 inhibitors, MLS-573151 and Casin, were used to scrutinize the process (46). The dosage of these inhibitors was also validated by observing significantly suppressive expression on WASP, the downstream molecular of Cdc42, which was displayed in Figure S4. As expected, the fluorescence intensities of phagocytosed beads (Figure 3B) or bacteria (Figure 3C) in hemocytes, taken as a measure of phagocytosis efficiency, were markedly reduced in granulocytes treated with MLS-573151 or casin compared to that in the control group (one-way ANOVA; statistical significance determined at *p* < 0.05). Specifically, the phagocytosis index of MLS-573151-treated groups was about 3.0-fold lower than that of the control, for either case of internalized FITC-labeled beads or FITC-labeled bacteria. In the case of casin, the inhibition of phagocytosis index dropped from 62 to 35% for internalized FITC-labeled beads and 69 to 35% for internalized FITC-labeled bacteria. Nevertheless, the Cdc42 inhibitors harbored little effect on phagocytosis index of hyalinocytes. These results show that both Cdc42 inhibitors (casin and MLS-573151) could effectively inhibit the phagocytic ability of granulocytes, thereby confirming the important role of Cdc42 in regulating granulocytes function.

### Regulatory Roles of Transcription Factor FOS in Transcriptional Activation of Granulocyte-Specific Genes

**Figure 4:**
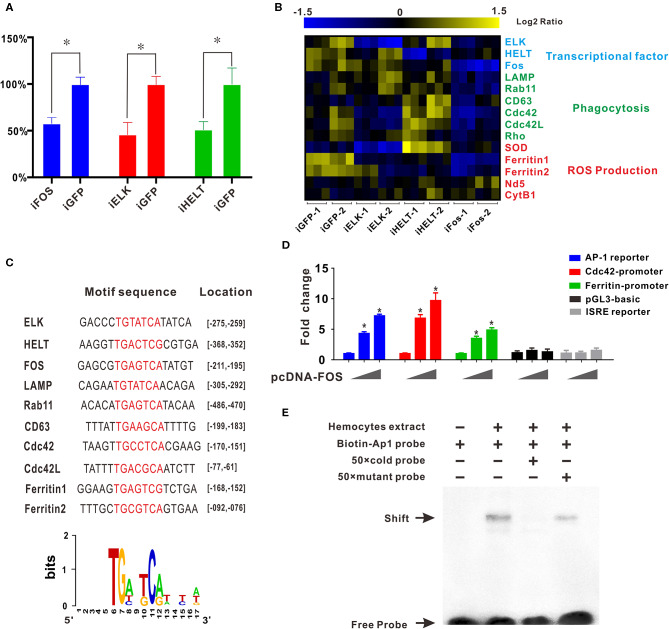
Transcription factor Fos regulates transcriptional activation of granulocyte-specific genes. **(A)** Inhibition rate of RNA interference of three transcription factors (Fos, ELK, and HELT) *in vivo*. *Blue panel*, interference of Fos; *red panel*, interference of ELK; *green panel*, interference of HELT. **(B)** mRNA expression levels of immunity-related genes after knockdown of three transcription factors (ELK, HELT, and Fos). Detected genes were classified into three groups based on their potential functions: transcription factors, phagocytosis, and ROS production. Colors from yellow to blue indicate range of log_2_ ratio values (in descending order); yellow color indicates high expression level and blue color indicates low expression level. **(C)** Transcription factor binding site prediction software identified an AP-1 site that was highlighted in promoter element sequence of some immunity-related genes. Specific nucleotides were marked with red color. **(D)** Relative luciferase activity by expression of Fos plasmid for the luciferase reporter genes, AP-1 reporter Luc (*blue panel*), Cdc42 promoter reporter Luc (*red panel*), Cdc42l promoter reporter Luc (*green panel*), pGL3-basic vector (*black panel*), and ISRE-luc reporter (*gray panel*) in HEK293T cells. Plasmid pcDNA-Fos were added in gradient concentration (0, 150, and 300 ng). *p < 0.05. **(E)** AP-1 probe was labeled with biotin and incubated with *C. gigas*-extracted hemocyte proteins. Unlabeled specific competitor sequences (cold probe) were used in a 50-fold surplus over labeled target.

Transcription factor network has long been considered to orchestrate hematopoiesis or cell differentiation, guiding gene expression to become a more specific type of cell. To further elucidate the regulatory functional mechanisms of granulocyte-specific genes, several important and predominantly expressed transcription factors in granulocytes, including *ELK, HELT*, and Fos, were identified and knocked down by RNAi to explore their potential function in hemocytes (Figure 4A). Interference of single transcription factors had been successfully achieved by RNAi techniques, and qPCR analysis of gene mRNA expression after RNAi showed that knockdown of Fos could effectively reduce the expression of essential phagocytosis related genes, such as LAMP, Rab11, CD63, Cdc42, etc. (required for phagocytosis) and reduce the expression of ferritin1 and ferritin2 (implicated in ROS production). However, knockdown of other transcription factors had no apparent impact on the expression of these genes (Figure 4B). Subsequently, we obtained the promoter sequences of these Fos target genes and established a common transcription factor binding site, the AP1 binding site TGANTCA (Figure 4C), which further validated the regulatory role of Fos in these target genes, suggesting the regulatory role of Fos in phagocytosis and ROS production.

Furthermore, *in silico* prediction showed that multiple AP-1 binding sites located in the proximal promoters of Cdc42 and Cdc42l (Figures S5, S6). To assess whether Fos could direct transcriptional activation of Cdc42, the AP-1 reporter (*blue panel*), Cdc42-promoter reporter (*red panel*), Cdc42-like (Cdc42l)-promoter reporter (*green panel*), and two negative control vectors (pGL3 vector, *black panel*, and ISRE reporter, *gray panel*) were used to detect Fos transcriptional activity. In this study, we observed that Fos could enhance activation of the AP-1 reporter by 7.5-fold with a transfection dose of 300 ng, and this activation was found to be dose-dependent (Figure 4D, *blue panel*). No obviously activated effect was observed in two negative control groups, indicating the specifically transcriptional activity of Fos on Cdc42 promoter and AP-1 site. Moreover, Fos also produced enhancing effects on the Cdc42-promoter with an increase of more than 10-fold (Figure 4D, *red panel*) and also activated ~6.0-fold on activity of Cdc42l-promoter reporters (Figure 4D, *green panel*), suggesting that Fos participated in the Cdc42 and Cdc42l regulatory signaling networks. These results suggest that activation of Fos strongly facilitated the gene expression involving the AP1 gene, Cdc42, and Cdc42-like promoter sequences.

To validate functional specificity of AP-1 binding site in hemocytes, EMSA was performed to probe interactions between extracted hemocyte proteins and transcription factor binding sites and AP1 binding site. As shown in Figure 4E, a clear shift band became observable upon incubation of the biotin-labeled AP-1 probe with extracted hemocyte proteins (Lane 2), with respect to a negative control (Lane 1). Meanwhile, high concentrations of a competitive probe (unlabeled AP-1 probe) resulted in abolition of binding of extracted hemocyte proteins to the probe (lane 3), whereas addition of excess mutant probe (TAANTTA) had no effect on the binding of extracted hemocyte protein to the AP-1 probe (lane 4). Collectively, these results lend strong support to the establishment of Fos as a pivotal regulator in functional differentiation of hemocytes, via the AP-1 binding site.

## Discussion

Hemocytes have been long recognized for their vital roles in host immune response, which encompass precisely regulated processes such as recognizing, locating, ingesting, transporting, and digesting foreign particles (48). Traditionally, mollusk hemocytes are broadly classified into granulocytes and hyalinocytes on the basis of their morphological heterogeneity in invertebrates (13, 14, 28). Our observations via light microscopy and TEM also suggest this in *C. gigas*. Granulocytes are distinguished from other hemocytes by a defining anatomical feature, their large and ample cytoplasmic granules, while hyalinocytes have hyaline cytoplasm and silky appearance. Cytoplasmic granules in granulocytes are loaded with a variety of hydrolases designed to destroy phagocytosed foreign particles (38, 49, 50). The capacities for phagocytosis and ROS production of granulocytes in *C. gigas* are evidently greater than those of hyalinocytes, as demonstrated in this study, in agreement with previous reports (26). Although the molecular mechanisms underlying hemocyte phagocytosis and bacteria clearance have been extensively studied (51), the molecular determinants governing functional differentiation of hemocytes remain largely unknown. Sorting of hemocyte subpopulations coupled to transcriptomic analysis is ideal for advancing a more comprehensive view on functional differentiation between granulocytes and hyalinocytes in *C. gigas*. In our study, two hemocyte subpopulations profiled through four groups analyzed (50 samples per group) could be distinguished on the basis of granule content (SSC), while cell size (FSC) varied continuously over a wide range (19). Cells of different size classes were sorted and subsequently subjected to transcriptomic analysis for global identification of DEGs in granulocytes. This proposed strategy is advantageous for exploring the molecular driving functional heterogeneity and diversity in granulocytes, in conjunction with morphological and functional observations.

Granulocytes of *C. gigas* exhibit a number of important morphological and functional characteristics (48, 52, 53) and employ distinct molecular components and related signaling pathways, which make them stand out from hyalinocytes. Transcriptomic analysis here highlights a large array of DEGs, nearly all of which were prominently upregulated in granulocytes. These high expressed genes are themselves predominantly found in hemocytes in comparison with other tissues and are functionally involved in at least seven signaling pathways. Among these pathways, regulation of actin cytoskeleton, phagosome, and endocytosis, and lysosome were most relevant to phagocytosis (54, 55), corroborating the strong capacity of granulocytes for engulfing microbes. Extensive works have previously established that the mitogen-activated protein kinase (MAPK) pathway plays critical roles in the regulation of a wide range of cellular processes including cell proliferation, differentiation, migration, senescence, and apoptosis (56). Of note, MAPK activity has been shown to respond to hematopoietic cytokines and growth factors, promoting the regulation of hematopoiesis (57). Furthermore, MAPK in mollusks was reported to be activated by various forms of extracellular stimulation and operate as a key regulator in immunity (58, 59). Our DEG analysis reveals that MAPK participated in granulocyte activities to regulate functional activities, prior to the hyalinocytes, suggesting its involvement in *C. gigas* functional differentiation of hemocytes.

Notably, the DEGs in granulocytes formed a regulatory network with Cdc42 as a core member, which orchestrates the regulation of actin cytoskeleton, phagosome and endocytosis, and MAPK signaling pathway. Our results strongly support the notion that the phagocytosis capacity of granulocytes was dampened in the presence of Cdc42 inhibitors. Cdc42 is a small GTP-binding protein of the Rho family, which act as molecular switches between inactive GDP and active GTP-bound states to regulate actin remodeling (60, 61). In addition, another GTP-binding protein, Rho, was found to be closely connected with Cdc42 and Cdc42l in our pathway networks. Rho was the first Rho family protein to be implicated in the regulation of actin remodeling in response to extracellular signals (60). Thus, phagocytosis in granulocytes appears to be driven by Cdc42 as a regulatory switch in actin-dependent events. In this context, it is noteworthy that Cdc42-dependent actin dynamics has been suggested to control cell maturation, secretory activity (62), endocytosis (63), and cell adhesion to the extracellular matrix (64). Previously, dendritic cell maturation was reportedly accompanied by substantial rearrangements of actin cytoskeleton, leading to an enhanced transport of vesicle to the cell surface (65). In this current study, we provided fresh evidence on the significance of Cdc42 as a regulator to give rise to functional granulocytes in *C. gigas*, although the precise mechanistic details with which Cdc42 acts to regulate granulocyte phagocytosis remain to be elaborated.

Clearly, functional differentiation is regulated by various endogenous and exogenous factors, among which, transcriptional regulators play a pivotal role in modulating cell development, leading to functional heterogeneity and diversity between cells (66). Indeed, four transcription factors were identified in granulocyte-specific genes, including HELT, ELK, Fos, and microphthalmia-associated transcription factor (MITF). However, literature-based evidences supported that HELT, ELK, and Fos have strong potentials in regulation of hematopoiesis (67–69). Furthermore, AP-1 (activator protein 1, Fos/Jun) transcription factors have been shown to be versatile regulators in the functional development of hematopoietic cell lineages, including monocytes/macrophages (70), granulocytes (71, 72), megakaryocytes (73), mastocytes (74), and erythroid lineages (75). AP-1 computes extracellular signals into expression programs of specific target genes that have AP-1 binding sites in their promoter or enhancer regions (76). In agreement with this idea, knockdown of expression of the AP-1 transcription factor Fos in *C. gigas* diminished the expression levels of genes involved in phagocytosis (LAMP, Rab11, CD63, Cdc42, Cdc42l, and Rho) and ROS production (SOD, ferritin1, ferritin2, Nd5, and CytB1). Evidence from our promoter analysis indicates that all of these downstream genes harbor the AP-1 binding site (TGTATCA). AP-1 binding site is commonly identified in the genes required for phagocytosis and ROS production. Additionally, a network of DEGs illustrated actively engaged signaling pathways, with Cdc42 and Cdc42l being a core regulator of pathway network in functional differentiation of hemocytes. By means of a dual-luciferase reporter assay, it was demonstrated that *C. gigas* Cdc42 and Cdc42l are, in turn, also tightly regulated by the expression of Fos. These observations thus offer compelling evidence for the functional significance of transcription factor Fos in transcriptional activation of granulocyte-specific genes and then consequently promote the functional differentiation of hemocytes in *C. gigas*. However, due to the lack of powerful transgene techniques or available oyster hemocytes cell lines so far, it is still difficult to perform the rescue experiment by reactivation of Cdc42 with Fos knockdown in oyster hemocyte, which limited us to obtain directive evidence of Fos/Cdc42 guiding the functional differentiation of granulocytes.

**Figure 5:**
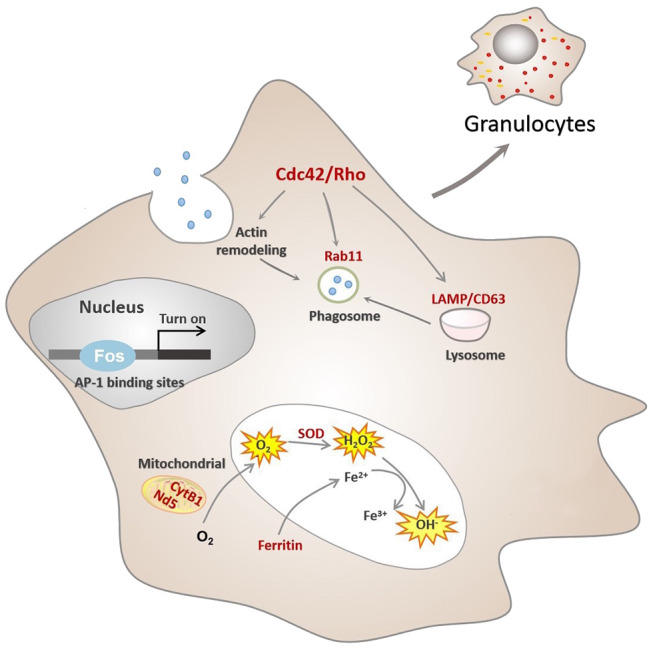
Conceptualization of functional differentiation of hemocytes in *C. gigas*. During functional differentiation, expression of the transcription factor Fos rises, which turns on transcription by binding to the AP-1 binding site of target genes, marked in red color. These target genes contribute to functional activities of granulocytes, including ROS production and phagocytosis. Cdc42/Cdc42l, the core regulators of granulocytes pathway network, were also transcriptionally controlled by Fos to promote actin rearrangement during phagosome formation in granulocytes, ultimately facilitating phagocytosis.

Hemocytes constitute a crucial cellular component of *C. gigas* immunity, though the processes underpinning their formation (or hematopoiesis) in mollusks are far from completely understood. It has been generally agreed that invertebrate granulocytes are more phagocytic than agranulocytes (28, 77) and show similarities to the human granulocyte–monocyte macrophage lineage, particularly vertebrate monocytes/macrophages. The mononuclear phagocyte lineage undergoes a multistep process toward maturation from bone marrow precursors to tissue macrophages (MAC) via circulating blood monocytes (MO) (30, 78, 79). In this study, it is established that an intricate assembly of molecular switches is at work to control several hallmarks of granulocyte biology and regulate functional differentiation of hemocytes in a marine invertebrate, *C. gigas*. For simplicity, we propose a developmental sequence of *C. gigas* hemocytes, as shown in Figure 5. Although hematopoiesis is common to all animals with circulatory systems, the precise mechanisms and outcomes of hematopoietic events are quite different due to genetic complexity and interactions across different species. Hence, this study has put forward a novel molecular picture on functional differentiation of hemocytes in *C. gigas*, which may ultimately facilitate our understanding of how immune defense systems evolve and operate in higher-order and more recent phyla.

## Data Availability Statement

The datasets generated for this study can be found in the NCBI Sequence Read Archive database under the accession number PRJNA591303.

## Ethics Statement

Experiments in this study were conducted with approval from Experimental Animal Ethics Committee, South China Sea Institute of Oceanology, Chinese Academy of Sciences, China.

## Author's Note

Experiments in this study followed standard biosecurity and institutional safety procedures.

## Author Contributions

FM conceived the study, analyzed the transcriptome data, performed flow cytometry, RNAi, imaging and EMSA experiments, and prepared the manuscript. YL performed the transcriptomic analysis and protein annotation. XZ performed the inhibition experiment. MH and DX performed the motif analysis and dual-luciferase reporter assay. ZX and JL prepared the hemocytes. YZ, N-KW, and ZY conceived and interpreted the data, and prepared the manuscript. All authors drafted the work, revised it critically for important intellectual content, and approved the final version.

## Conflict of Interest

The authors declare that the research was conducted in the absence of any commercial or financial relationships that could be construed as a potential conflict of interest.

## Supplementary Material

Figure S1 **(A,B)** KEGG pathway analysis.

Figure S2 Q-PCR validation of key DEGs.

Figure S3 Correlation plot of DEGs.

Figure S4 Relative expression level of WASP after Cdc42 inhibition.

Figure S5 Cdc42 promoter.

Figure S6 Cdc42L promoter.

Table S1 Sequencing data.

Table S2 Alignment efficiency statistics.

Table S3 DEG analysis.

Table S4 DEGs in common.
