# Supplementary figures and images for: Transcriptomic Evidence Reveals the Molecular Basis for Functional Differentiation of Hemocytes in a Marine Invertebrate, Crassostrea gigas
Source: Front Immunol. 2020 May 27;11:911. doi: 10.3389/fimmu.2020.00911 (PMC7269103; doi:10.3389/fimmu.2020.00911)

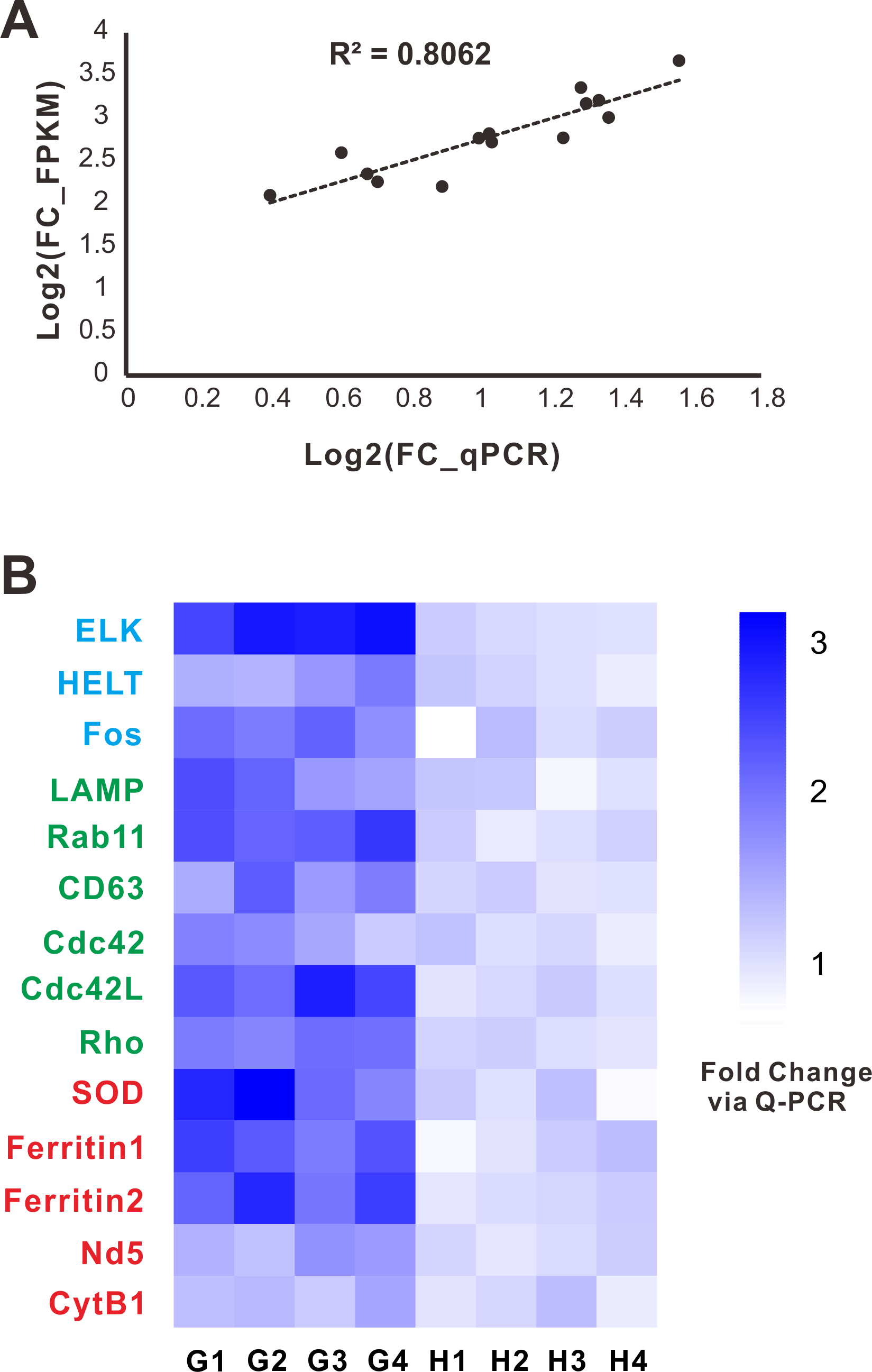

Supplement: Figure S1 — (A,B) KEGG pathway analysis. [file Image_1.tif]

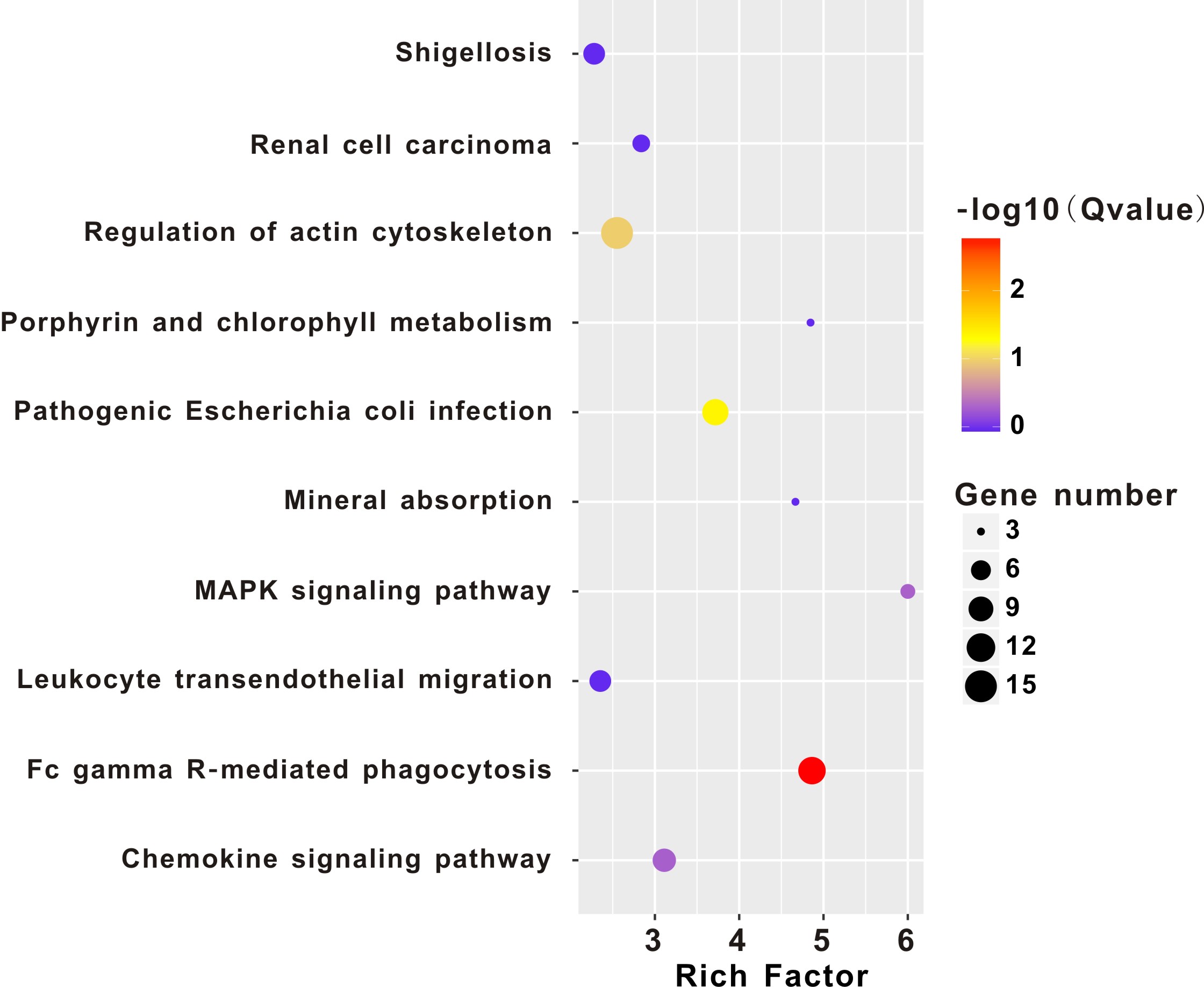

Supplement: Figure S2 — Q-PCR validation of key DEGs. [file Image_2.jpeg]

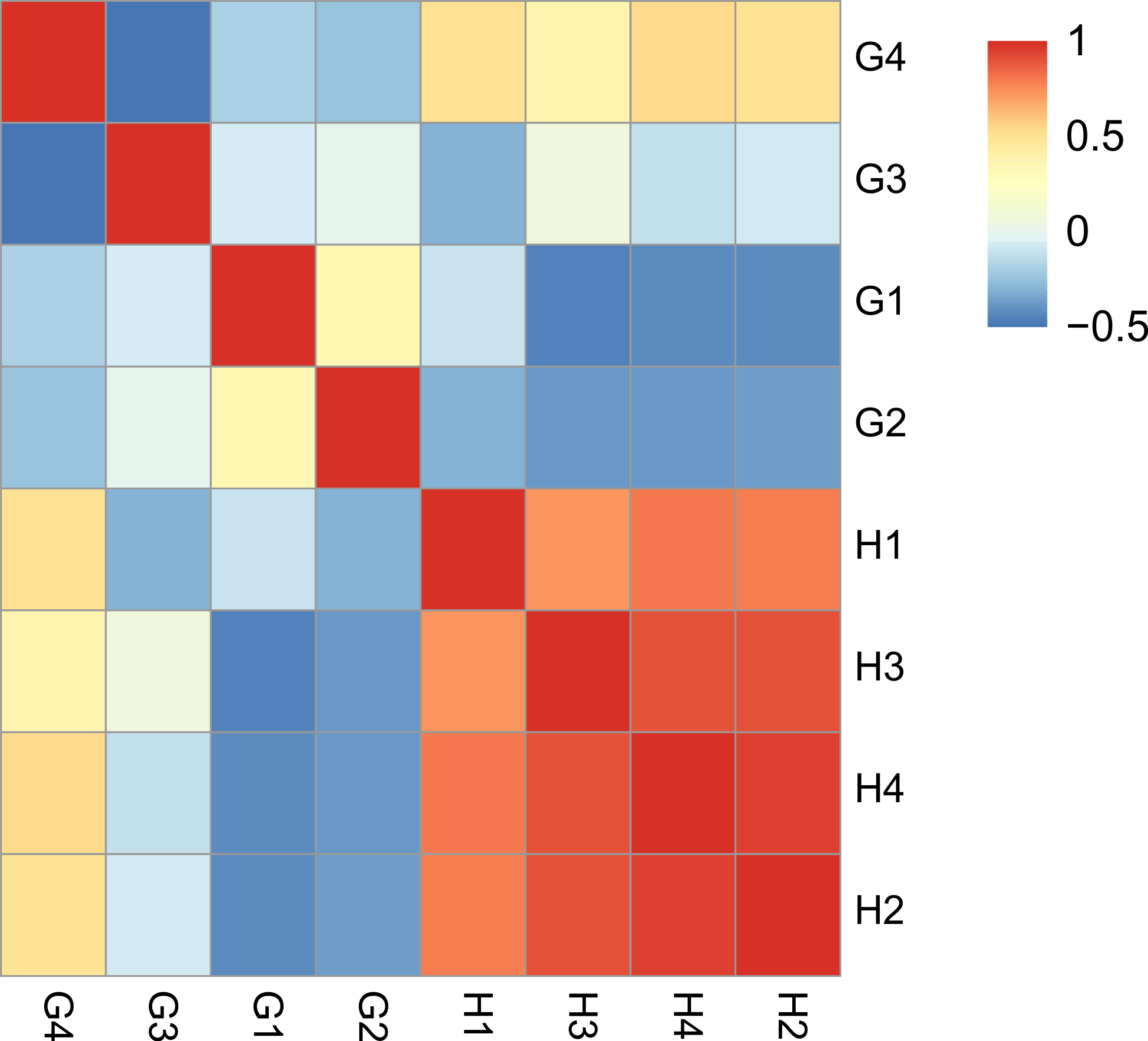

Supplement: Figure S3 — Correlation plot of DEGs. [file Image_3.TIF]

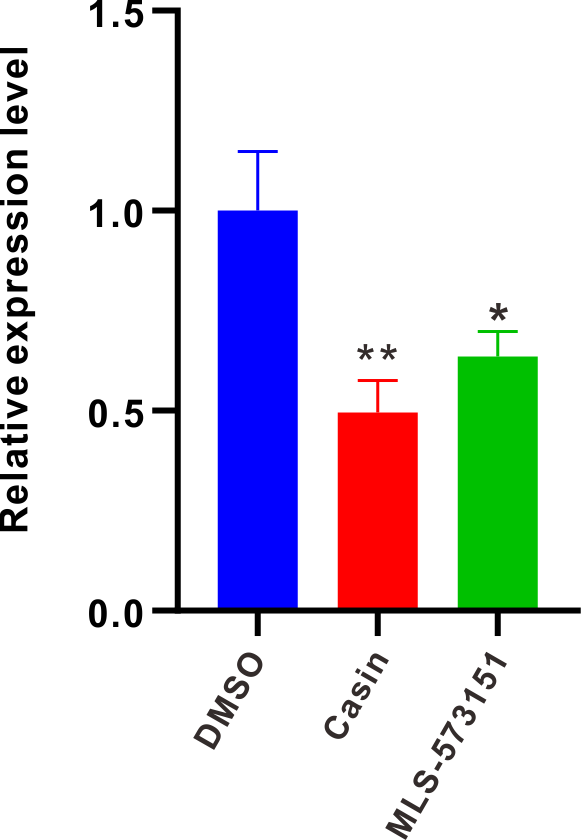

Supplement: Figure S4 — Relative expression level of WASP after Cdc42 inhibition. [file Image_4.TIF]

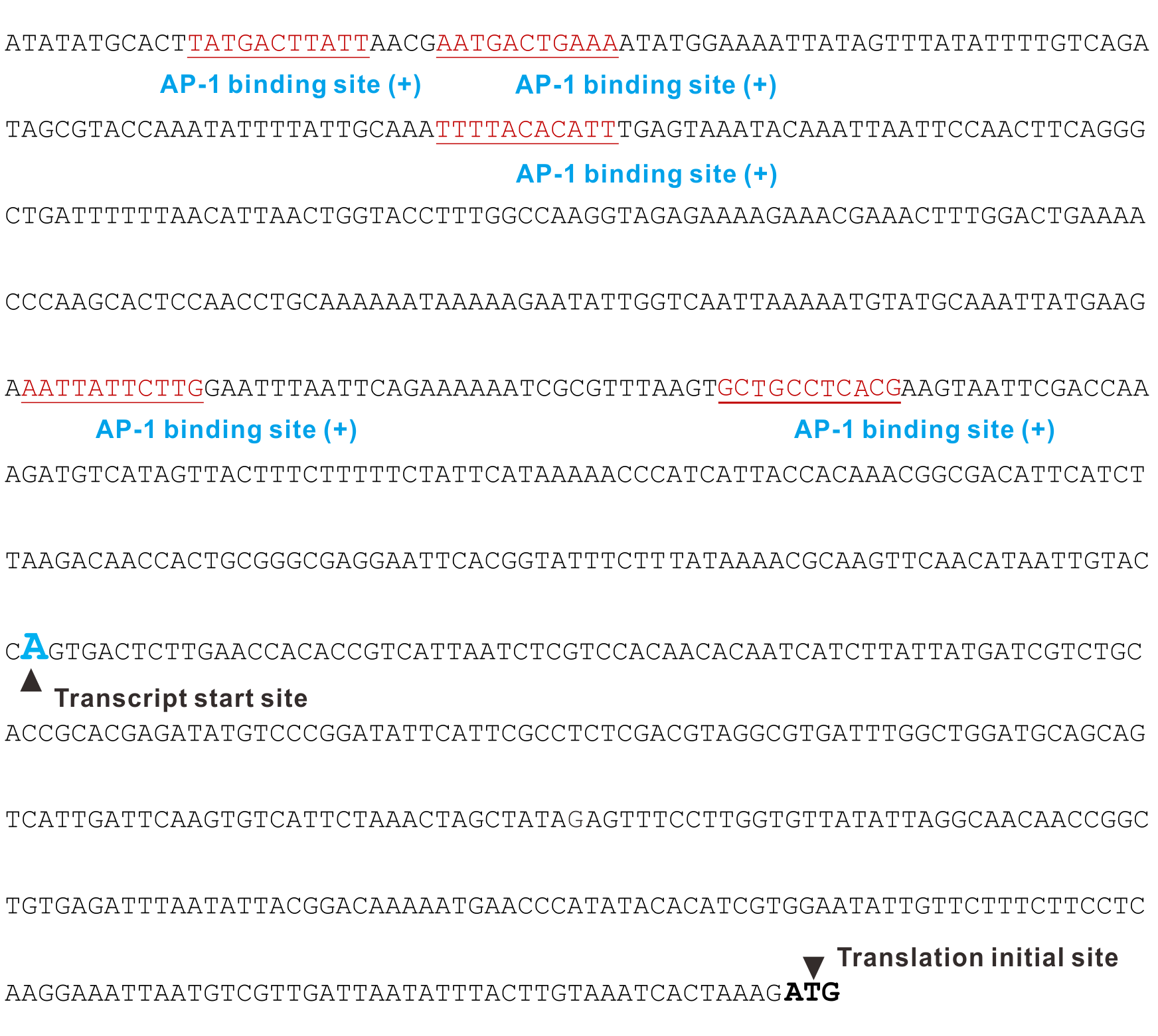

Supplement: Figure S5 — Cdc42 promoter. [file Image_5.TIF]

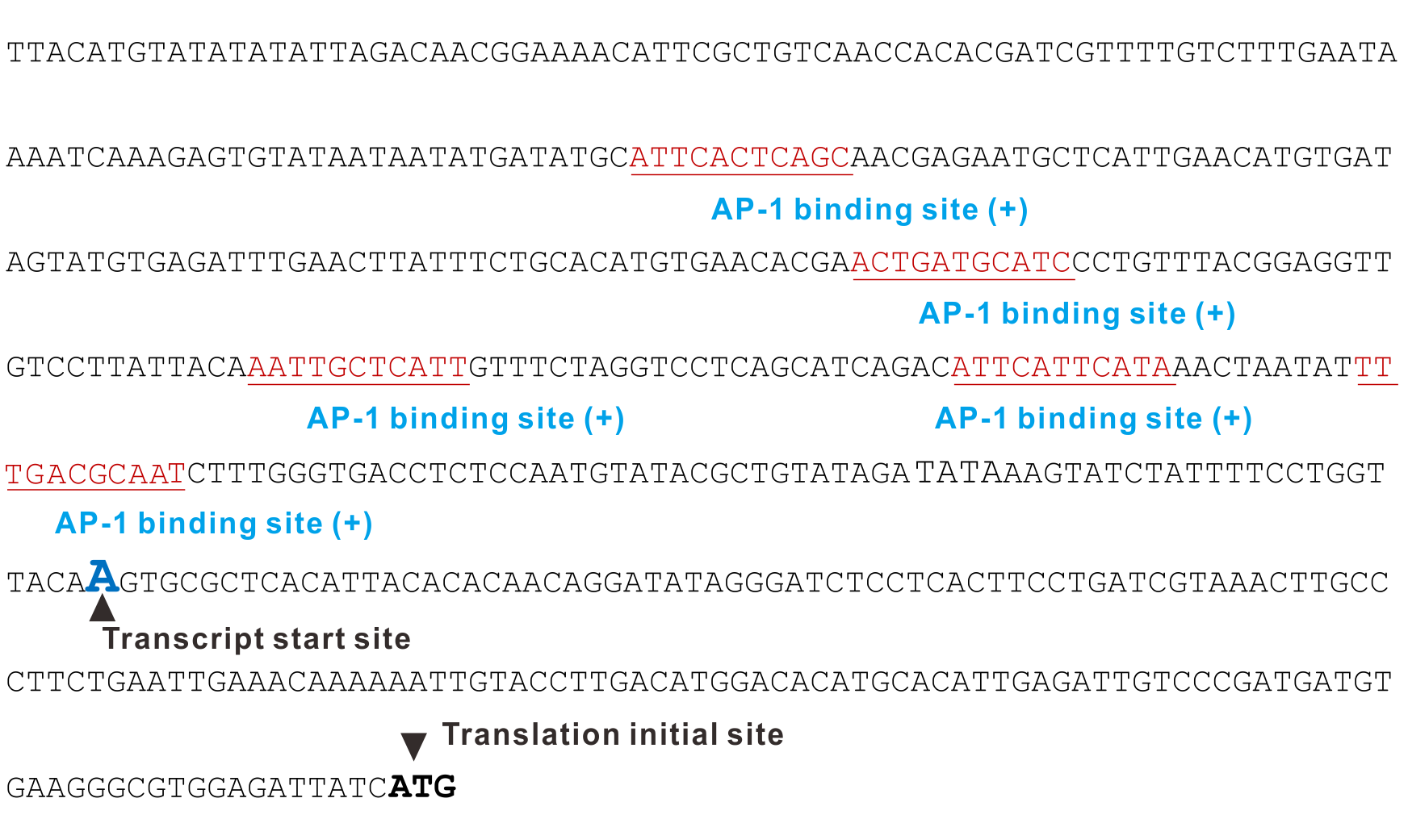

Supplement: Figure S6 — Cdc42L promoter. [file Image_6.TIF]
