# Supplementary material for: Transcriptomic Evidence Reveals the Molecular Basis for Functional Differentiation of Hemocytes in a Marine Invertebrate, Crassostrea gigas
Source: Front Immunol. 2020 May 27;11:911. doi: 10.3389/fimmu.2020.00911 (PMC7269103; doi:10.3389/fimmu.2020.00911)
Supplement: Table S1 — Sequencing data. [file Table_1.DOCX]

**Table S1 Sequencing data**

Through sequencing, we obtained a total of 125,076,161M sequencing reads. In the statistical evaluation of sequencing quality values, the base Q30 was more than 92.32%. Generally Q30 is more than 80%, the sequencing quality can be considered very reliable. The specific statistical evaluation of sequencing data can refer to the following table:

| **Samples** | **Groups** | **Total reads** | **Total nucleotides (bp)** | **GC percentage** | **Q30 percentage** |
| --- | --- | --- | --- | --- | --- |
| G1 | R1 | 15,426,774 | 786,383,336 | 38.02 | 93.28 |
| G2 | R2 | 13,013,001 | 663,339,791 | 40.10 | 92.89 |
| G3 | R3 | 9,737,117 | 496,480,767 | 42.03 | 92.39 |
| G4 | R4 | 9,846,846 | 502,035,666 | 42.06 | 92.48 |
| H1 | R1 | 13,228,322 | 674,321,771 | 39.30 | 93.28 |
| H2 | R2 | 10,061,933 | 512,885,512 | 38.45 | 93.42 |
| H3 | R3 | 13,116,287 | 668,710,743 | 40.75 | 92.69 |
| H4 | R4 | 12,150,005 | 619,378,002 | 42.22 | 92.32 |
