# Supplementary material for: Transcriptomic Evidence Reveals the Molecular Basis for Functional Differentiation of Hemocytes in a Marine Invertebrate, Crassostrea gigas
Source: Front Immunol. 2020 May 27;11:911. doi: 10.3389/fimmu.2020.00911 (PMC7269103; doi:10.3389/fimmu.2020.00911)
Supplement: Table S2 — Alignment efficiency statistics. [file Table_2.DOCX]

**Table S2 Alignment efficiency statistics**

The results of the sequence alignment of the sample sequencing data and the selected reference genome are shown in the following table:

| **Samples** | **Total reads** | **Mapped reads** | **Unique mapped reads** | **Multiple mapped reads** |
| --- | --- | --- | --- | --- |
| G1 | 15,426,774 | 6,802,341(44.09%) | 3,940,853(57.93%) | 2,861,488(42.07%) |
| G2 | 13,013,001 | 5,832,580(44.82%) | 3,080,271(52.81%) | 2,752,309(47.19%) |
| G3 | 9,737,117 | 5,645,266(57.98%) | 1,929,014(34.17%) | 3,716,252(65.83%) |
| G4 | 9,846,846 | 5,611,851(56.99%) | 3,184,716(56.75%) | 2,427,135(43.25%) |
| H1 | 13,228,322 | 5,664,748(42.82%) | 3,530,744(62.33%) | 2,134,004(37.67%) |
| H2 | 10,061,933 | 3,827,243(38.04%) | 2,941,683(76.86%) | 885,560(23.14%) |
| H3 | 13,116,287 | 6,168,081(47.03%) | 3,452,649(55.98%) | 2,715,432(44.02%) |
| H4 | 12,150,005 | 5,482,211(45.12%) | 3,192,997(58.24%) | 2,289,214(41.76%) |

Total reads: the number of all reads; the number of reads in the brackets for the total number of reads; Mapped reads: the number of reads on the reference genome; Unique mapped reads: the number of reads unique to the alignment; Multiple mapped reads: The number of reads with more than one position.
